# Physical Unclonable Functions with Hyperspectral Imaging System for Ultrafast Storage and Authentication Enabled by Random Structural Color Domains

**DOI:** 10.1002/advs.202401983

**Published:** 2024-06-18

**Authors:** Xiaofeng Lin, Quhai Li, Yuqi Tang, Zhaohan Chen, Ruilian Chen, Yingjuan Sun, Wenjing Lin, Guobin Yi, Quan Li

**Affiliations:** ^1^ School of Chemical Engineering and Light Industry Guangdong University of Technology Guangzhou 510006 P. R. China; ^2^ Guangdong Provincial Laboratory of Chemistry and Fine Chemical Engineering Jieyang Center Jieyang 515200 China; ^3^ Institute of Advanced Materials and School of Chemistry and Chemical Engineering Southeast University Nanjing 211189 China; ^4^ Key Laboratory for Polymeric Composite and Functional Materials of Ministry of Education Sun Yat‐sen University Guangzhou 510275 China; ^5^ Materials Science Graduate Program Kent State University Kent OH 44242 USA

**Keywords:** cellulose nanocrystal, encrypted storage, hyperspectral imaging, physical unclonable function, structural color domain

## Abstract

Physical unclonable function (PUF) is attractive in modern encryption technologies. Addressing the disadvantage of slow data storage/authentication in optical PUF is paramount for practical applications but remains an on‐going challenge. Here, a highly efficient PUF strategy based on random structural color domains (SCDs) of cellulose nanocrystal (CNC) is proposed for the first time, combing with hyperspectral imaging system (HIS) for ultrafast storage and authentication. By controlling the growth and fusion behavior of the tactoids of CNC, the SCDs display an irregular and random distribution of colors, shapes, sizes, and reflectance spectra, which grant unique and inherent fingerprint‐like characteristics that are non‐duplicated. Based on images and spectra, these fingerprint features are used to develop two sets of PUF key generation methods, which can be respectively authenticated at the user‐end and the manufacturer‐front‐end that achieving a high coding capacity of at least 2^2304^. Notably, the use of HIS greatly shortens the time of key reading and generation (≈5 s for recording, 0.5–0.7 s for authentication). This new optical PUF labels can not only solve slow data storage and complicated authentication in optical PUF, but also impulse the development of CNC in industrial applications by reducing color uniformity requirement.

## Graphical abstract

Based on the random structural color domains on the surface of cellulose nanocrystal composite films, two sets of physical unclonable function encryption methods have been developed to meet the anti‐counterfeiting needs of both the end user and the manufacturer. The use of a hyperspectral camera, which can acquire the reflectance spectra of a 480 × 480 pixels in ≈5 s, enables ultra‐fast storage and authentication.

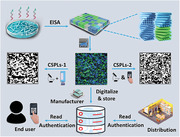

## 1 Introduction

Counterfeiting is a pervasive global issue impacting industries like pharmaceuticals, food, and luxury goods, leading to substantial economic losses and potential health risks.^[^ ^1^ ^]^ To counteract the suppress and combat the escalating problem of counterfeit goods, rising challenge of counterfeit goods, researchers have developed various anti‐counterfeiting materials. These include digital including digital watermarks,^[^ ^2^ ^]^ diffraction gratings,^[^ ^3^ ^]^ luminescent inks,^[^ ^4^ ^]^ surface wrinkle materials,^[^ ^5^ ^]^ and structured color materials.^[^ ^6^ ^]^ Despite these advancements, criminals with adequate technology can easily replicate and alter these materials, rendering them ineffective for reliable protection of goods. Consequently, there is a pressing need for a dependable anti‐counterfeiting solution to effectively tackle the escalating issue of counterfeit products globally.

The Physical unclonable function (PUF) security technology, characterized by its non‐duplicability and unpredictability emerges as emerges as labeling and digital storage.^[^ ^7^ ^]^ PUFs are essentially random formations of disordered microscopic or nano‐structures, uniquely identifiable like fingerprints.^[^ ^7^, ^8^ ^]^ Once created, replicating a PUF security label is challenging even for the manufacturer, let alone for third‐party offenders, significantly enhancing the product's security dimension.^[^ ^9^ ^]^ Over the last decade, research has focused on various PUF materials and fabrication methods, including silicon‐based or transistor‐based PUFs,^[^ ^10^ ^]^ surface morphology‐based PUFs,^[^ ^11^ ^]^ bioinformatics‐based PUFs like DNA,^[^ ^12^ ^]^ and optical PUFs.^[^ ^13^ ^]^ Optical PUFs, in particular, have seen active research since the pioneering work by Pappu et al.^[^ ^14^ ^]^ with classifications based on their “response” in the “Challenge Response Pair”, such as fluorescent,^[^ ^15^ ^]^ phosphorescent,^[^ ^16^ ^]^ Raman,^[^ ^17^ ^]^ scattering,^[^ ^17^, ^18^ ^]^ polarized^[^ ^19^ ^]^ and structured color^[^ ^9^, ^20^ ^]^ PUFs. However, Raman and fluorescence PUFs face challenges with stability, undergoing irreversible structural changes and fluorophore decomposition upon exposure to excitation light.^[^ ^9^ ^]^ These optical PUFs often suffer from slow key input and time‐consuming authentication processes. In particular, the acquisition and storage of large amounts of spectral information often takes tens of minutes or more. Moreover, their authentication requires complex, expensive instrumentation and expert operation, posing barriers to end‐user authentication needs. Furthermore, some optical PUFs may lack biocompatibility, limiting their application. Thus, addressing the of slow encoding input, inconvenient reading and poor stability in optical PUF anti‐counterfeiting labels, and developing a green PUF anti‐counterfeiting label remain a challenge.

Structural color, unlike traditional pigments or dyes that rely on light absorption, is a purely physical phenomenon, produced without any pigment. It offers environmental friendliness, long‐term color stability without damage or deformation of the material structure, color angle dependence, and resistance to photobleaching.^[^ ^21^ ^]^ Bio‐based materials with exceptional optical properties, such as cellulose nanocrystal (CNC) extracted from cotton and other plants, present as a renewable nanomaterial. CNC is noted for its biocompatibility, biodegradability, and low cost, and has the ability to self‐assemble into iridescent photonic films with a chiral nematic phase structure.^[^ ^6^, ^22^ ^]^ However, advances in the controllable tuning of nanoscale CNC self‐assembly, scaling up the fabrication of CNC optical films remains a challenge, which hinders the wider application of CNC optical films.^[^ ^23^ ^]^ Integrating unclonable properties into CNC iridescent color films can mitigate the stringent color uniformity requirements in industrial applications of CNC. It also paves the way for the emergence of a new generation of green, rapid‐storage PUF anti‐counterfeiting optical labels. This innovation could address the issues of slow code input and poor stability encountered with optical PUFs. In response to the aforementioned challenges of optical PUFs, we propose utilizing the structural color of CNC films as the “response” in our new generation of optical PUFs.

In this study, we have developed an efficient anti‐counterfeiting strategy utilizing random structural color domains (SCDs) in PUF combined with a hyperspectral imaging system (HIS) for ultrafast data storage and authentication. These optical PUF labels, namely CNC structural color PUF labels (CSPLs) are synthesized through evaporation ‐induced self‐assembly (EISA) using a blend of CNC, polyvinylpyrrolidone‐K30 (PVP‐K30), and glycerol (Gly). The resulting structural color is non‐uniform, and comprises SCDs of varying colors, mainly due to defects from the fusion of tactoids during CNC nanorods assembly. The SCDs size can be adjusted by controlling the evaporation rate of the co‐mixing suspension, and their color by the PVP‐K30 content. We established the first PUF encryption scheme based on the position, shape, and color of SCDs achieving a coding capacity of at least 2^2500^. This encryption can be authenticated rapidly using affordable stereomicroscopes or smartphones with attached microscopes, facilitating quick consumer‐level authentication (0.5–0.7 s for a database of 1000 labels). Additionally, a second PUF encryption scheme utilizing the reflectance spectra of SCDs, with a coding capacity of 2^2304^, is implemented using the HIS. This system can store the reflectance spectra of 480 × 480 pixels on the label in ≈5 s. In a database of 1000 labels, authentication is achievable within 0.5–0.7 s, depending on computer performance. The HIS streamlines the generation of spectra‐related keys and simplifies the front‐end storage and authentication process for manufacturers. The rapid storage and excellent authentication speed of these green CSPLs position them as a potent solution for information security, significantly contributing to anti‐counterfeiting efforts and promoting industrial applications of CNC.

## 2 Results and Discussion

### 2.1 The Co‐Assembly of CNC/PVP‐K30/Gly and their Nanostructures

Renewable CNC was extracted from alkali‐treated cotton pulp via concentrated sulfuric acid hydrolysis.^[^ ^6d^ ^]^ Atomic force microscopy (AFM) analysis revealed that CNC has a rod‐like structure, averaging 155 ± 25 nm in length and 12 ± 6 nm in diameter (Figure S1, Supporting Information). Zeta potential indicates that both the CNC suspension and the CNC/polyvinylpyrrolidone‐K30/glycerol (CPG) suspension form well‐dispersed aqueous colloidal systems,^[^ ^6g^ ^]^ providing a solid foundation for the preparation of photonic films through EISA of these suspensions (Figure S2, Supporting Information). PVP‐K30 and Gly, known for their good biocompatibility, are frequently used as excipients in pharmaceutical formulations.^[^ ^24^ ^]^ In this study, PVP‐K30 as the principal filler, and Gly were introduced to form extra hydrogen bonding to enhance the film's flexibility. Therefore, we used CPG suspension to obtain CSPLs by EISA. Additionally, X‐ray diffraction (XRD) analysis of CNC film, CSPLs, and alkali‐treated cotton paper revealed that the acid hydrolysis and the addition of PVP‐K30 did not alter the crystal structure of CNC (Figure S3, Supporting Information). As shown in Figure S4 (Supporting Information), The pure CNC films and CSPLs in Figure 2g were characterized by Fourier transform infrared spectroscopy (FT‐IR). The results showed that hydrogen bond interaction was formed between the hydroxyl group of glycerol, the hydroxyl group of CNC and the amide group of PVP‐K30, and a hydrogen bond cross‐linking network was constructed.^[^ ^22d^ ^]^

As shown in Figure S5 (Supporting Information), when observed under a stereomicroscope, the surface of the CNC film is distributed with discontinuous blue “islands” and transparent regions. These “islands” are termed SCDs. When the filler is filled, the SCDs on the surface of CSPLs are predominantly blue and green, with transparent and some cyan SCDs also distributed across on the film surface.

**Figure 1:**
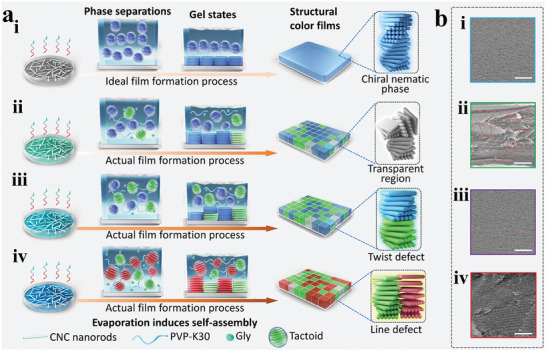
a) Schematic illustration of EISA of CNC suspensions with different PVP‐K30 and Gly contents. The processes include the ideal EISA of pure CNC i) and the actual EISA of CPG suspensions filled with 15%, 25%, and 45% of CNC mass of PVP‐K30, respectively ii–iv), where Gly constitutes 10% of the mass of the corresponding PVP‐K30. b) Scanning electron microscopy (SEM) images of CNC film and CSPLs sections, the red dashed lines are the demarcation lines of different domains, i) chiral nematic phase structure without defect (scale bar, 2 µm), ii) transparent region (scale bar, 8 µm), iii) twist defect (scale bar, 2 µm), and iv) line defect (scale bar, 600 nm).

The appearance of randomly distributed SCDs is mainly caused by defects generated by CNC in the EISA process (successively entering the phase separation and gel state process).^[^ ^25^ ^]^ As the suspension undergoes phase separation through evaporation, CNC nanorods aggregate to form small tactoids, which possess axial orientation and a periodic structure.^[^ ^23d^ ^]^ During this process, the tactoids are capable of continuous and free movement. While also adjusting the helical axis orientation, which tends to be perpendicular to the substrate, the small tactoids further contact and fuse due to the presence of disordered‐ordered interfacial tension. As the water in the suspension further evaporates, the system enters a gel state, leading to kinetic trapping. Here, the orientation and spatial position of the tactoids are locked, and the pitch can only contract along the direction of the helix axis.^[^ ^25^, ^26^ ^]^ **Figure** **1**a shows the brief EISA process of CNC suspension and CPG suspensions. Ideally, the tactoids remain perpendicular to the substrate during evaporation, merging into a perfect chiral nematic phase macrostructural domain (Figure 1a‐i,b‐i). However, in both CNC suspension and co‐mixtures of CNC with other filling media, not all tactoids maintain the same direction, periodic bandgap or size after entering the gel state. This leads to the formation of line defect and stop band twist defect after the fusion of tactoids with different orientations or periodic bandgaps.^[^ ^26^, ^27^ ^]^ Scanning electron microscopy (SEM) characterization of the CNC film or CSPLs cross section, confirmed the presence of twist defect and line defect. Figure 1b‐iii and Figure S6 (Supporting Information) show the twist defects in the film cross section, mainly present between two chiral nematic structures with different layer spacings in the same vertical plane, affecting the abrupt change in the refractive index period. Notably, the presence of twist defects may cause double peaks in the reflection spectra of cyan SCDs (Figure S13, Supporting Information), indirectly suggesting that some cyan domains display cyan light due to the mixing of blue and green light. Figure 1b‐iv and Figure S7 (Supporting Information) clearly illustrate how line defects separate two distinct chiral nematic structures with different layer spacings. These structures correspond to the green and red SCDs within the film's internal makeup. Based on the SEM results and prior research,^[^ ^26^, ^27^ ^]^ these defects are likely the boundaries or edges of different SCDs, impacting the size of these domains and the reflected color. Furthermore, the distribution of PVP‐K30 within the CSPLs may vary in different regions, contributing to the presence of multicolored SCDs in the film.

The transparent region on the surface of the CSPLs also influences the information distribution on the film surface. We demonstrated that this region's transparency primarily arises from the chaotic distribution structure of its corresponding section or the insufficiently large pitch. The polarizing microscopy (POM) and AFM analyses (Figure S8, Supporting Information) suggests that the surface and interior of both regions are distributed with chiral nematic phase structures. SEM image of the transparent regions (Figure 1b‐ii) reveals the presence of several domains within the same cross‐section, each with a vertical thickness of only 1 µm. These domains are too thin to reflect enough structural color to be captured by an optical microscope,^[^ ^27a^ ^]^ and therefore appear transparent. Consequently, the transparency of these regions can be attributed to the lack of sufficiently thick domains within the corresponding sections. Notably, an ordered distribution is also observed by SEM in some transparent region sections (Figure S9, Supporting Information). However, the pitch (*P*) of these regions was only measured to be only ≈230 ± 20 nm, and according to Bragg's formula:

(1) $$\lambda_{max}=\;n_{avg}Psin\theta$$

where *λ*_max_ is the maximum reflection peak wavelength, *n*_avg_ represents the average refractive index of the medium, which is 1.54, and *θ* represents the angle of incidence, which is 90°,^[^ ^22a^ ^]^ *λ*_max_ is 354 nm, which is in the ultraviolet region invisible to the naked eye.

### 2.2 Size and Color Regulation of SCDs

**Figure 2:**
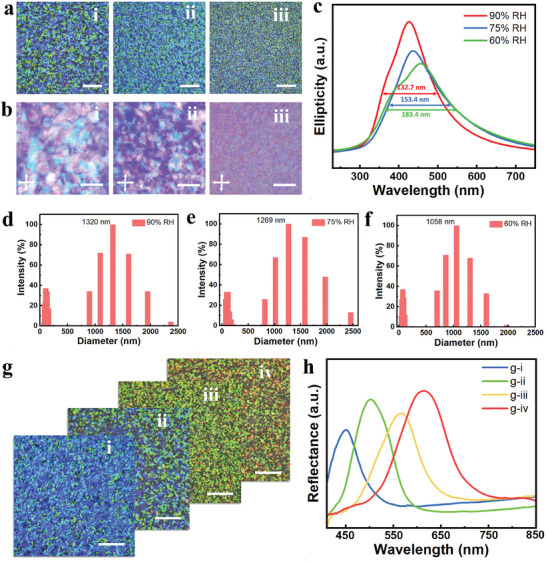
a) Images of CSPLs (scale bars, 500 µm) obtained in an evaporative environment at 20 °C and relative humidities of i) 90% RH, ii) 75% RH, and iii) 60% RH, respectively. b) POM images (scale bars, 100 µm) of the corresponding CSPLs in (a). c) circular dichroism (CD) spectra of the corresponding CSPLs in (a). d–f) Statistics of particle size distribution after evaporation of the CPG suspensions mass fraction from 3.2 wt.% to 4.2 wt.% at 20 °C and relative humidities of 90%, 75% and 60%, respectively. g) CSPLs with varied color distributions of SCDs are fabricated, i‐iv) images of CSPLs produced using PVP‐K30 with filler quantities of 15%, 25%, 35%, and 45% of the CNC mass (scale bars, 500 µm). Furthermore, the filler amount of Gly is 10% of the corresponding PVP‐K30 mass. The reflectance spectra are displayed in (h).

The size of SCDs on CSPLs is primarily controlled by adjusting the evaporation rate during the EISA stage. The evaporation rate can be adjusted by changing the relative humidity (RH). It is known that the SCDs on the films surface are mainly caused by defects generated from the fusion of tactoids during the EISA stage. Tactoids in the phase‐separated suspension tend to align perpendicularly to the Petri dish substrate.^[^ ^26a^ ^]^ However, when the phase‐separated suspension is poured into a Petri dish, tactoids orientation is initially disorderly, taking time to align perpendicular to the substrate. Once the suspension enters the gel state, a kinetic trapping phenomenon occurs. The positions and orientations of the tactoids are locked, and the pitch can only shrink along the helical axis direction. Therefore, in the EISA stage, the evaporation rate is regulated to control the time it takes for the suspension to reach the gel state, and thus the time available for the tactoids to reorient themselves. The slower the evaporation rate, the greater the number of tactoids oriented perpendicular to the Petri dish substrate, thus reducing the chance of defect formation, and fusion to obtain larger tactoids. In **Figures** **2**a‐i–iii and S10a‐i–iii (Supporting Information), a series of CSPLs and pure CNC films obtained by controlling the evaporation temperature to 20 °C and the relative humidity at 90% RH, 75% RH, and 60% RH, respectively, were shown, and the sizes of their SCDs decreases with the decrease in relative humidity. In Figure 2a‐i–iii, the average sizes of the SCDs of these three CSPLs were measured to be 220, 120, and 20 µm using a stereomicroscope. The corresponding POM images of these CSPLs and CNC films (Figure 2b‐i‐iii; Figure S10b‐i–iii, Supporting Information) also show the decrease of the domain size with the decrease of the relative humidity. Furthermore, the full width at half maximum of the CD spectra in films correlates with the films' defect density.^[^ ^28^ ^]^ CD characterizations (Figure 2c; Figure S10c, Supporting Information) were performed on these CSPLs and CNC films. As we expected, the full width at half maximum of their CD spectra increased with decreasing relative evaporation humidity of EISA stage. The full width at half maximum increased from 132.7 nm (90% RH) to 183.4 nm (60% RH) in the CSPLs, and full width at half maximum increased from 98.5 nm (90% RH) to 154.6 nm (60% RH) in the CNC films. Finally, the particle size distributions of each suspension after evaporation of the mass fraction from 3.5 to 4.5 wt.% at 90% RH, 75% and 60% RH, respectively, were measured using a laser light scattering system. As shown in Figure 2d–f and Figure S10d–f (Supporting Information), the particle size distribution of the largest agglomerates reduced with decreasing relative humidity for both CPG suspensions and CNC suspensions. This also illustrates the effect of evaporation rate at the EISA stage on the size of the SCDs after film formation.

The color distribution of SCDs on CSPLs is closely related to their internal periodic structure. The pitch value of the nematic phase structure can be tuned by varying the content of PVP‐K30 in the CSPLs, consequently regulating the color of SCDs on the film surface. As shown in Figure 2g, CSPLs with different PVP‐K30 fillings were prepared at 20 °C, and 90% RH. The PVP‐K30 fillings were 15%, 25%, 35%, and 45% of the CNC mass, respectively, with Gly being 10% of the corresponding PVP‐K30 filling mass. SCDs of the blue and green group (Figure 2g‐i,ii), the green and yellow group (Figure 2g‐iii), and the green and red group (Figure 2g‐iv) were sequentially acquired. Figure 2h, the reflection spectra of these CSPLs measured by an optical fiber spectrometer indicate that the highest peaks of their reflection spectra correspond to wavelengths of 447, 503, 566, and 614 nm, in sequence.

By modulating the color and size of the SCDs in CSPLs, we created a solid foundation for the preparation of CSPLs for use in PUF labels.

### 2.3 PUF Based on Structural Color

**Figure 3:**
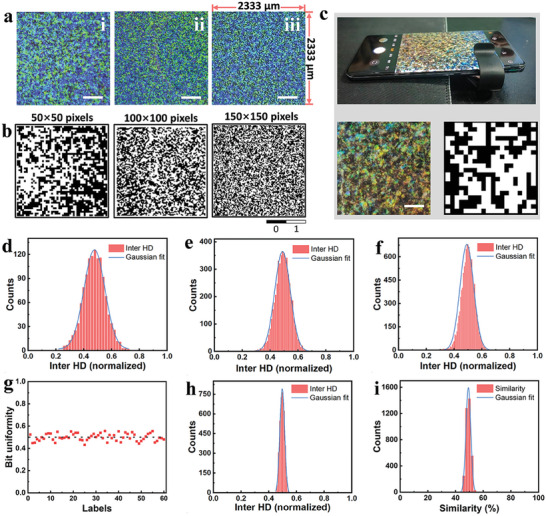
CSPLs‐1 key generation and PUF parameter extraction. a) Optical images for PUF analysis (scale bars, 500 µm). b) The corresponding CSPLs‐1 key for the labels in (a). c) Optical image information is taken using a smartphone and a portable microscope and digitized to get a 20 × 20 pixels CSPLs‐1 key (scale bar, 300 µm). The inter HD of the CSPLs‐1 key for 50 × 50, 100 × 100 and 150 × 150 pixels are shown in d–f), respectively. g) Bit uniformity of 60 different CSPLs‐1 of 50 × 50 pixels, and the corresponding inter HD shown in h) and similarity statistic shown in i).

The random distribution of SCDs on the surface of the CSPLs, including their structural color, shape, position, and size, endows the CSPLs with inherent, unique, and fingerprint‐like unclonable properties. Therefore, based on these fingerprint characteristics, we have developed the first PUF encryption technique (CSPLs‐1). Initially, this approach involves capturing optical images of the CSPLs via a stereomicroscope, and then using a MATLAB program to convert the data from optical images into binary code, thereby acquiring the CSPLs‐1 keys. The CSPLs with different sizes of SCDs obtained by evaporation at 90% RH, 75% RH, and 60% RH, are shown in **Figure** **3**a‐i‐iii. The label size is 2333 µm × 2333 µm. The MATLAB program maps the blue SCDs and transparent regions as black pixels (0), and the green and cyan SCDs as white pixels (1), in the optical images of the CSPLs. For the different sizes of SCDs, their resolution sizes were reduced to 50 × 50, 100 × 100, and 150 × 150 pixels, respectively. Finally, the CSPLs‐1 keys were obtained, as shown in Figure 3b. The encoding capacities of these keys are 2^2500^, 2^10000^, and 2^22500^, respectively. To demonstrate the unclonable property of CSPLs‐1, we computed the security of labels using several metrics, including bit uniformity, uniqueness, randomness, and similarity. The bit uniformity values computed were 0.4766, 0.4884, and 0.4964 for the labels corresponding to 50 × 50, 100 × 100, and 150 × 150 pixels, respectively. These values, being close to the ideal value of 0.5, indicate these strong bit uniformity in the labels. The uniqueness of the labels was quantified in terms of the normalized Inter hamming distance (Inter HD). Figure 3d–f shows the statistical distribution of Inter HD for CSPLs‐1 of 50 × 50, 100 × 100, and 150 × 150 pixels. The mean values of the Inter HD are 0.5200, 0.5556, and 0.4912, respectively, all close to the ideal value of 0.5. Each Inter HD graph exhibits a normal distribution, indicating better uniqueness in the labels, and hence their unclonable capability.^[^ ^17a^ ^]^ Furthermore, we conducted the same procedure for 60 50 × 50 pixels CSPLs‐1 keys. Figure 3g displays the bit uniformity statistics of these labels, with a distribution range of 0.5 ± 0.06. The Inter HD for these 60 labels was calculated, yielding a mean value of 0.5000, aligning with a normal distribution as evidenced in Figure 3h. Further, the similarity index of these labels (the percentage of identical pixel counts between two labels^[^ ^17e^ ^]^) was found to be 51.29%, approaching the ideal value of 50% (Figure 3i). Investigating the randomness of CSPLs‐1 involved calculating the entropy of the specified 60 50 × 50 pixels CSPLs‐1, as presented in Figure S11 (Supporting Information). The entropy values, nearing the ideal of 1, with an average of 0.9961 and a minimum of 0.9905, indicating the randomness of CSPLs‐1. These findings suggest that CSPLs‐1, leveraging unique features like structural color, demonstrate notable uniformity, randomness, and distinctiveness. Such attributes indicate that CSPLs‐1 could be exceptionally effective in anti‐counterfeiting applications.

In practical applications, CSPLs‐1, with their fingerprint features, can be visualized using cost‐effective and portable readout devices, as depicted in Figure 3c. This process is straightforward and does not requires complex instruments. By using a smartphone and a portable microscope, an image can be captured and the optical information of CSPLs‐1 extracted. Subsequently, this data can be digitized to produce a 20 × 20 pixels security key. The use of inexpensive and portable readout devices, makes CSPLs‐1 easily accessible to a broader range of end users. Eliminating the need for costly instruments and specialized expertise, CSPLs‐1 become more practical and economical for anti‐counterfeiting applications.

### 2.4 Digital Label Transformation of Spectral Information

SCDs of CSPLs provide rich reflectance spectral information, adding another layer of fingerprint properties to the CSPLs. By extracting and converting these spectral details into PUF keys, we establish a second layer of PUF encryption (CSPLs‐2), enhancing the security of anti‐counterfeit labels. Nevertheless, the small size of SCDs, typically ranging from tens to hundreds of micrometers, makes it challenging to directly and accurately locate and collect their spectral information using conventional fiber optic spectrometers, and acquisition of large amounts of spectral data is also very time‐consuming. To address this issue, we incorporated a hyperspectral camera with a stereomicroscope to establish a HIS (Figure S12, Supporting Information). The hyperspectral camera, a detection device integrateing spectral acquisition and target imaging, uses imaging spectroscopy to cover the same target area in continuous spectral bands. This allows the device to quickly provide detailed spectral information for each pixel of the image.^[^ ^29^ ^]^ The HIS enables precise location of the spectral information corresponding to a pixel point of CSPLs‐2. In this study, the HIS captured an image measuring 2333 µm × 2333 µm, containing 480 × 480 pixels. The spectral information of all 480 × 480 pixels was collected simultaneously, and the extraction process was completed in ≈5 s.

The reflectance spectra of a single pixel within blue SCDs, green SCDs, cyan SCDs, transparent regions, and red SCDs of the CSPLs were acquired and compared using the HIS (Figure S13a,b, Supporting Information). SEM characterizes the corresponding cross sections of each SCDs. According to the results of Figure S13 (Supporting Information), the selective reflectance spectra of different SCDs form the basis for the development of CSPLs‐2.

**Figure 4:**
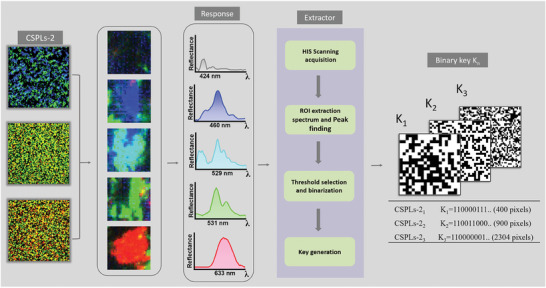
CSPLs‐2 keys generation and PUF parameter extraction.

**Figure 5:**
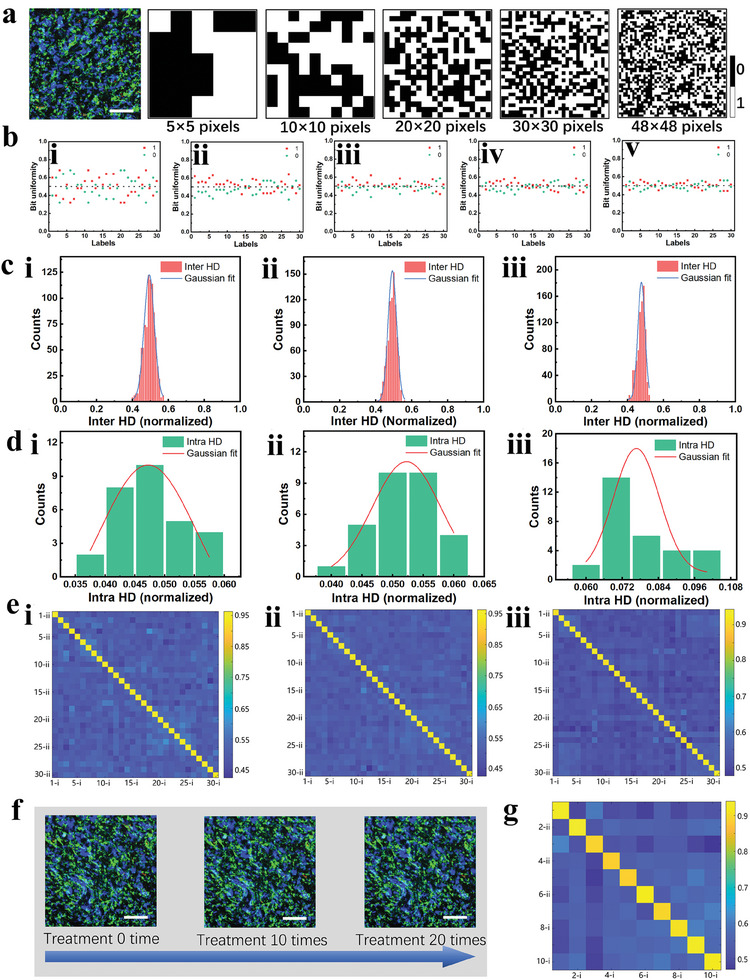
The CSPLs‐2 keys are shown in a) (scale bar, 500 µm). b) Bit uniformities of i) 5 × 5 pixels, ii) 10 × 10 pixels, iii) 20 × 20 pixels, iv) 30 × 30 pixels and v) 48 × 48 pixels. The Inter HD in c) and Intra HD in d) distribution of 30 CSPLs‐2 keys of 20 × 20, 30 × 30 and 48 × 48 pixels, and the corresponding similarity statistics graph are shown in (e). f) Optical image of the same region of CSPLs after 20 cycles of high humidity and high temperature treatment (scale bars, 500 µm). g) After processing 10 CSPLs as in f), 10 CSPLs‐2 keys (48 × 48 pixels) are generated, and then the similarity statistic image is computed before and after processing.

The flow diagram in **Figure** **4** illustrates the process of generating CSPLs‐2 keys when challenged by excitation. After the HIS collects the spectral information of the 480^2^ pixels of the label, Regions of Interests (ROIs) of 5 × 5, 10 × 10, 20 × 20, 30 × 30, and 48 × 48 pixels are set in the ENVI software (Figure S14, Supporting Information). These ROIs are used to read reflectance spectral information of the label at various resolutions. A MATLAB program then uses 489 nm as a threshold wavelength, mapping pixels whose wavelength corresponding to the highest intensity of the reflection peak is less than the threshold as “0” and those greater than or equal to the threshold as “1”. The mapping data of the reflection spectra are then converted into CSPLs‐2 keys of 5 × 5, 10 × 10, 20 × 20, 30 × 30, and 48 × 48 pixels (**Figure** **5**a). This procedure is repeated 30 times to produce 30 distinct security keys. Figure S15 (Supporting Information) shows the 48 × 48 pixels CSPLs‐2 key corresponding to 30 labels. Figure S16 (Supporting Information) compares a 50 × 50 pixels CSPLs‐1 key with a 48 × 48 pixels CSPLs‐2 key for one label. Bit uniformity testing on various resolution keys associated with these security keys reveals a gradual improvement in bit uniformity, approaching the ideal value of 0.5, as the number of pixels increases, as shown in Figure 5b‐i–v. Consequently, we focused on uniqueness, repeatability and randomness for CSPLs‐2 keys of 20 × 20, 30 × 30, and 48 × 48 pixels. To assess the uniqueness of the CSPLs‐2 keys, we calculated the normalized Inter HD for different CSPLs‐2 and the normalized Intra Hamming Distance (Intra HD) for the same CSPLs‐2 keys. Gaussian fits were applied to both sets of HD results to obtain curves. As depicted in Figure 5c‐i–iii, the normalized Inter HD of 30 CSPLs‐2 are calculated. The mean values for the Inter HD of 20 × 20, 30 × 30, and 48 × 48 pixels CSPLs‐2 keys are 0.4934, 0.4921, and 0.4791, respectively, with corresponding standard deviation of 0.0673, 0.0562, and 0.0199. These distributions exhibit a normal pattern. Intra HDs are calculated by taking two measurements of all 30 CSPLs‐2 using the HIS. The resulting normalized mean values for Intra HDs are 0.0472, 0.0523, and 0.0776, with corresponding standard deviations of 0.0186, 0.0131, and 0.0180, respectively (Figure 5d‐i–iii). These mean values of CSPLs‐2 Inter HD and Intra HD are close to the ideal values of 0.5 and 0, respectively, indicating high levels of uniqueness and repeatability. Meanwhile, Figure 5e shows the similarity statistics obtained from two measurements of 30 CSPLs‐2 keys. The comparison results indicate high similarity for the same PUF label measured twice, while different PUF labels exhibit lower similarity. Gaussian fits were also conducted on the calculated intra HD and inter HD values to identify the overlap region and estimate authentication mode thresholds (Figure S17, Supporting Information). The thresholds for 20 × 20, 30 × 30, and 48 × 48 pixels CSPLs‐2 are 0.148, 0.138, and 0.266, respectively. These thresholds determine the false‐negative rate, representing the probability of authentication failure for initially authenticated CSPLs‐2. The coding capacities of 20 × 20, 30 × 30, and 48 × 48 pixels keys are 2^400^, 2^900^, and 2^2304^, respectively.

The entropies of the 20 × 20, 30 × 30, and 48 × 48 pixels CSPLs‐2 are all close to the ideal value of 1, with average entropies of 0.9936, 0.9944, and 0.9761, respectively (Figure S18, Supporting Information). The high entropy indicates that CSPLs‐2 exhibit a high degree of randomness. Furthermore, the National Institute of Standards and Technology Randomness Test Suite (NIST SP 800–22) is a statistical method used for assessing the randomness of binary sequences of arbitrary length.^[^ ^30^ ^]^ Therefore, 30 binary sequences of 20 × 20 pixels CSPLs‐2 (12000 bits) were submitted for validation. The computational results demonstrate that these binary sequences successfully pass the NIST randomness test suite (Table S1, Supporting Information).

### 2.5 Stability of CSPLs

In practical applications, environmental stability is crucial for PUF labels. To evaluate the stability of CSPLs, we performed a series of environmental stress tests on 10 labels. These labels were exposed to 99% relative humidity and subsequently placed in an oven at 80 °C, repeating 20 times. Figure 5f shows that the SCDs on the CSPLs surface remain stable even after enduring a series of environmental stress tests. Subsequently, these 10 labels were analysed as 48 × 48 pixels CSPLs‐2 keys both before and after the environmental stress tests, and similarity statistics were calculated. The analysis results were consistent with the encoding capability of CSPLs (Figure 5g). These findings indicate that CSPLs maintain strong stability under environmental fluctuations. Figure S19 (Supporting Information) presents a comparison of changes before and after placing one CSPLs in a normal environment for 60 days. We also calculated the similarity of the corresponding 48 × 48 pixels CSPLs‐2 keys before and after this period, resulting in a similarity of 0.9084. This outcome suggests that the keys retain their stability over time, indicating the long‐term stability of CSPLs.

### 2.6 Rapid Storage and Authentication of CSPLs

The procedure to store the CSPLs‐1 keys is relatively straightforward. Initially, an optical image of the target area on a label attached to a product (for example, a pharmaceutical product, shown in Figure S20, Supporting Information) is captured using a stereomicroscope or a smartphone with a portable microscope. This optical image is then transformed into a binary key using MATLAB software and uploaded to the database (Figure S21, Supporting Information). For inputting CSPLs‐2 keys, the reflectance spectral information from the above labels is first scanned and read by the HIS. As demonstrated in Video S1 (Supporting Information), allows for scanning and acquiring sample information with a resolution of 480 × 480 pixels, in ≈5 s. As shown in Table S2 (Supporting Information) and Figure S22 (Supporting Information), the optical mapping of HIS is far superior to Raman mapping and other means. The ENVI software is then used to read the reflectance spectral information of 20 × 20, 30 × 30, and 48 × 48 pixels from a label sample as the basis for the PUF keys. This process takes ≈38 and 89 s and 5 min, respectively. Once the spectral data are acquired and analysed, the CSPLs‐2 keys are generated by setting a threshold and binarizing the spectral information in MATLAB software. The program generates the key in just a few seconds. The integration of the HIS with ENVI and MATLAB solves the problem of slow extraction and storage of large amounts of spectral information, significantly enhances the reading speed of the CSPLs‐2 keys, thereby increasing efficiency.

In practical applications, the rapid authentication of PUF authenticity is essential. We prepared 60 CSPLs‐1 (50 × 50 pixels) and generated 940 random binary keys of the same size using MATLAB. Subsequently, these 1000 keys were uploaded to the database. One of the 60 CSPLs‐1 was randomly selected, digitized to obtain its key, and then compared with the database. If a label with a similarity greater than 85% is found, it is considered authentic. The entire process, from the optical image to obtaining the authentication result, takes ≈12 s, with the average authentication matching time being ≈0.5–0.7 s (Video S2, Supporting Information). Similarly, 30 CSPL‐2 keys of 48 × 48 pixels were uploaded to the database along with 970 keys of the same size generated by MATLAB. The authentication process was similar to the CSPLs‐1, with the matching process taking ≈0.5–0.7 s (Video S3 and Figure S23, Supporting Information). The time required for the detailed database construction and authentication process is shown in Table S3 (Supporting Information). Our authentication speed is better than other PUF labels, as shown in Table S4 (Supporting Information) and Figure S24 (Supporting Information).

## 3 Conclusion

In this work, we probe the mechanism of formation of SCDs in CSPLs, where the color and size dimensions of SCDs can be artificially regulated. Building upon the SCDs of CSPLs, we devise PUF labels and established two sets of PUF key generation methods, CSPLs‐1 and CSPLs‐2. The CSPLs‐1 key is designed for easy readability and digitized by smartphones, providing a convenient means for consumers to verify authenticity of security labels. To overcome slow process of collecting spectral information bit by bit, which is characteristic of traditional optical PUF labels, we utilize the HIS to expedite the generation of CSPLs‐2 keys and reduce the processing time. By analyzing bit uniformity, randomness, and repeatability, we confirm that the keys generated by both PUF encryption methods meet the necessary standards. Additionally, the labels demonstrate stability under conditions of high relative humidity and high temperatures. In summary, we propose a green, non‐duplicable, easily identifiable, and practical anti‐counterfeiting method, offering an effective and feasible solution to combat counterfeiting.

## 4 Experimental Section

### Materials

Medical cotton was purchased from Xuzhou Kunpeng hygienic material Company (China). Sulfuric acid (AR, 95–98%) was purchased from Guangzhou Chemical reagent Factory (China). Polyvinylpyrrolidone K‐30 (AR) was purchased from Beijing Coolaibo Technology Co., LTD(China). Glycerol (AR) was purchased from Shanghai Macklin Biochemical Company (China).

### Preparation of Self‐Assembly CNC suspension

The CNC suspension was prepared by hydrolysis with concentrated sulfuric acid (64 wt.%). Briefly, medical cotton was first washed with 10 wt.% sodium hydroxide solution and then treated with pure water to neutral. The dried cotton fibers were hydrolyzed with 64 wt.% sulfuric acid and stirred at 45 °C for 60 min (sulfuric acid to cellulose mass ratio of 10). The reaction was terminated by adding 100 times the mass of pure water of sulfuric acid, and the initial suspension was allowed to stand and delaminate. After removing the supernatant by layering, the lower turbidity suspension was subjected to three high speed centrifugations at 10 000 rpm for 15 min each. At the end of centrifugation, the upper suspension was poured into a dialysis bag (12 000 MW) and dialyzed in pure water to pH = 3. Finally, the CNC suspension was concentrated in an oven at 55 °C to 3.2 wt.%.

### Preparation of CNC films

Three aliquots of 3.2 wt.% (3 g) CNC suspension with equal mass were successively placed in a constant temperature and humidity chamber to evaporate and assemble to obtain CNC films. The temperature was 20 °C, and the relative humidity was set at 90% RH, 75% RH, and 60% RH, respectively.

### Preparation of CSPLs

The first was the preparation of CSPLs with different sizes of SCDs. PVP‐K30 and Gly were added to three aliquots of CNC suspension (3.2 wt.%, 3 g), in which the mass of PVP‐K30 added to each suspension was 25% of CNC (0.096 g), and the mass of Gly added to each suspension was 10% of PVP‐K30. After mixing at room temperature for 6 h, the mixture was poured in polystyrene Petri dishes, and then evaporated into films in constant temperature and humidity chambers at 20 °C/90% RH, 20 °C/75% RH and 20 °C/60% RH, respectively. This was followed by the preparation of CSPLs with different sizes SCDs. PVP‐K30 and Gly were added to four aliquots of CNC suspension (3.2 wt.%, 3 g), where the mass of PVP‐K30 added to each suspension was 15%, 25%, 35%, and 45% of CNC (0.096 g), respectively, and the mass of Gly added to each aliquot was 10% of PVP‐K30. After mixing well at room temperature for 6 h, the mixture was poured in a polystyrene Petri dish, and then evaporated into a film in a constant temperature and humidity chamber at 20 °C and 90% RH.

### Characterization

The morphology of CNC nanorods was observed using AFM (Escalab 250Xi) to measure their size distribution. The microscopic morphology of the CNC film and CSPLs were observed by using an AFM. Zeta potential of the prepared CNC suspension and CPG suspension (0.1 wt.%) was carried out with a Malvern particle sizer (Zetasizer Nano ZS90). The particle size of the 4.2% suspensions was tested by laser light scattering system (BI‐ 200SM, Brookhaven, USA), and no dilution of the suspension was required. XRD spectra were obtained using an XRD diffractometer (D2 PHASER, Germany). The target material was chosen to be copper target with a wavelength of 0.15 418 nm, an accelerating voltage of 40 kV, an accelerating current of 40 mA, a scanning speed of 5° min^−1^, and a scanning angle of 5° – 50°. Infrared spectroscopy spectra of nanospheres of CNC film and CSPLs were obtained by using fourier transform infrared spectroscopy (FT‐IR, Thermo Scientific Nicolet 6700). CD spectra were measured by a BioTools MOS450 spectropolarimeter. POM Images of CNC films and CSPLs were captured by using an Olympus BX53 optical micrographic technique. SEM (Hitachi SU8220) was used to observe the cross sections of CNC film and CSPLs. The fluorescence spectra of CNC film were characterized by Fluormax – 4 fluorescence spectrophotometer (Horiba Scientific). The reflectance spectra of the CSPLs were obtained by using a spectrophotometer (Ocean Optics USB2000+), and the spectral information of the SCDs was collected by an HIS built with a hyperspectral camera (FigSpec FS23) and a stereomicroscope (Murzider, MSD9224).

### Label Alignment Method

First, design a perpendicular “T” alignment marker in the upper right corner of the target area on the label. the label was first placed on a rotatable X‐Y carrier platform, and then the system's microscope was adjusted to find the target area. Align the lower‐right edge of the “T” on the label with the upper‐left edge of the preview image window in the dedicated application by adjusting the X‐Y carrier platform. After alignment, the optical image of the label was taken or spectral information was obtained using HIS.

### PUF Performance Analysis

First, the generation of CSPLs‐1 key involves capturing optical images of CSPLs using a stereomicroscope or a portable microscope connected to a smartphone. Then, different‐sized images of SCDs were processed using MATLAB to perform binary thresholding, resulting in CSPLs‐1 key with varying resolutions. In the CSPLs‐1 key, a “1” represents the blue and transparent regions (the transparent areas appear black due to the black substrate), while a “0” represents the green and cyan regions. Second, the generation of CSPLs‐2 key involved extracting the spectral reflection information of each pixel using the HIS. The ENVI software was used to define ROIs of different sizes to extract corresponding spectral information. Afterwards, MATLAB was used to set thresholds and perform thresholding to obtain the CSPLs‐2 key. In the CSPLs‐2 key, a “1” represents spectral wavelengths greater than or equal to 489 nm, while a “0” represents spectral wavelengths less than 489 nm. Using these secure keys, MATLAB algorithms could be used to compute metrics such as bit uniformity, uniqueness, and repeatability.

### Stability Experiments

Ten labels were first placed in 99% RH for 30 min and then in an oven at 80 °C for 15 min, and the above operation was repeated 20 times. The optical pictures and spectral information of these labels were collected with the HIS on the 0th, 10th and 20th times, respectively. Afterwards, MATLAB was utilized to calculate the keys similarity between the pre‐treatment and post‐treatment.

## Conflict of Interest

The authors declare no conflict of interest.

## Supporting information

Supporting Information

Supplemental Video1

Supplemental Video2

Supplemental Video3

## Data Availability

The data that support the findings of this study are available in the supplementary material of this article.
