# Supplementary material for: Physical Unclonable Functions with Hyperspectral Imaging System for Ultrafast Storage and Authentication Enabled by Random Structural Color Domains
Source: Adv Sci (Weinh). 2024 Jun 18;11(31):2401983. doi: 10.1002/advs.202401983 (PMC11336904; doi:10.1002/advs.202401983)
Supplement: Supplementary file 1 — Supporting Information [file ADVS-11-2401983-s002.pdf]

ADVANCED SCIENCE
Open Access

Supporting Information

for *Adv. Sci.*, DOI 10.1002/advs.202401983

Physical Unclonable Functions with Hyperspectral Imaging System for Ultrafast Storage and Authentication Enabled by Random Structural Color Domains

Xiaofeng Lin, Quhai Li, Yuqi Tang, Zhaohan Chen, Ruilian Chen, Yingjuan Sun, Wenjing Lin, Guobin Yi and Quan Li**

Supporting Information

Physical Unclonable Functions with Hyperspectral Imaging System for Ultrafast Storage and Authentication Enabled by Random Structural Color Domains

Xiaofeng Lin †, Quhai Li †, Yuqi Tang †, Zhaohan Chen, Ruilian Chen, Yingjuan Sun, Wenjing Lin, Guobin Yi and Quan Li**

Prof. X. F. Lin, Q. H. Li, Z. H. Chen, Y. J. Sun, Prof. W. J. Lin, Prof. G. B. Yi
School of Chemical Engineering and Light Industry, Guangdong University of Technology, Guangzhou, 510006, P. R. China
E-mail: yigb@gdut.edu.cn;

Prof. X. F. Lin, Y. J. Sun, Prof. W. J. Lin, Prof. G. B. Yi
Guangdong Provincial Laboratory of Chemistry and Fine Chemical Engineering Jieyang Center, Jieyang, 515200, P. R. China

R. L. Chen
Key Laboratory for Polymeric Composite and Functional Materials of Ministry of Education, Sun Yat-sen University, Guangzhou, 510275, P. R. China

Y. Q. Tang, Prof. Q. Li
Institute of Advanced Materials and School of Chemistry and Chemical Engineering, Southeast University, Nanjing, 211189 China
Email: quanli3273@gmail.com

Prof. Q. Li
Materials Science Graduate Program, Kent State University, Kent, OH 44242 USA

[†] X. F. Lin, Q. H. Li and Y. Q. Tang contributed equally to this work.

Proof test for bit uniformity, uniqueness, repeatability, encoding capacity and randomness

Bit uniformity: In PUF key, bit uniformity refers to the degree to which the generated random bit sequence exhibits an equal frequency of "0" and "1". Specifically, if the random bit sequence generated by a PUF label shows a frequency of "0" and "1" that is close to 0.5, then the label possesses a high degree of bit unbiasedness. Uniformity is closely related to randomness.

$$Bit\ uniformity = \frac{1}{s}\sum_{l=1}^{s} k_l \tag{S1}$$

Where k_l is the lth binary bit of the key and s is the key size.

Uniqueness: Uniqueness can be quantified by Hamming inter distance (inter HD). Inter HD is the number of different responses between rows in a PUF label. The design formula for uniqueness is:

$$Uniqueness = \frac{2}{q(q-1)}\sum_{i=1}^{q-1}\sum_{j=i+1}^{q}\frac{HD(k_i, k_j)}{s} \tag{S2}$$

where q is the total number of rows in the binary code, $HD\ (k_i,\ k_j)$ is the Inter HD between two different lines in the binary code, k_i and k_j are s-bit keys for row i and row j in q, s is the total number of "0" and "1" in each row.

Repeatability: In order to test the feasibility of a reliable PUF label, the readout reproducibility of security keys from the same PUF label should also be tested. The repeatability of a PUF label indicates the ability to generate the same key after the same repeated challenge. Repeatability of PUF label can be defined as follows:

$$Repeatability = \frac{1}{m}\sum_{t=1}^{m}\frac{HD(k_i,k_{i,t})}{s} \tag{S3}$$

where s is the total number of pixels in each row, m is the number of measurements of the same PUF label, and $HD\ (k_i,\ k_i,\ t)$ is the Hamming intra distance (intra HD) between row i in the code.

Encoding capacity:

$$Encoding\ capacity = a^b \tag{S4}$$

where a is the number of responses ("0"and"1") of the PUF label's binarized code, and b is the total number of key pixels.

Randomness: The randomness of CSPLs can be evaluated by its entropy. The formula for uniqueness is:

$$Entropy = -[plog_2(p) + (1-p)log_2(1-p)] \tag{S5}$$

where p is the probability of "1" in CSPLs; the entropy is 1 when p is ideally 50 %.

Supplementary Figures and Tables

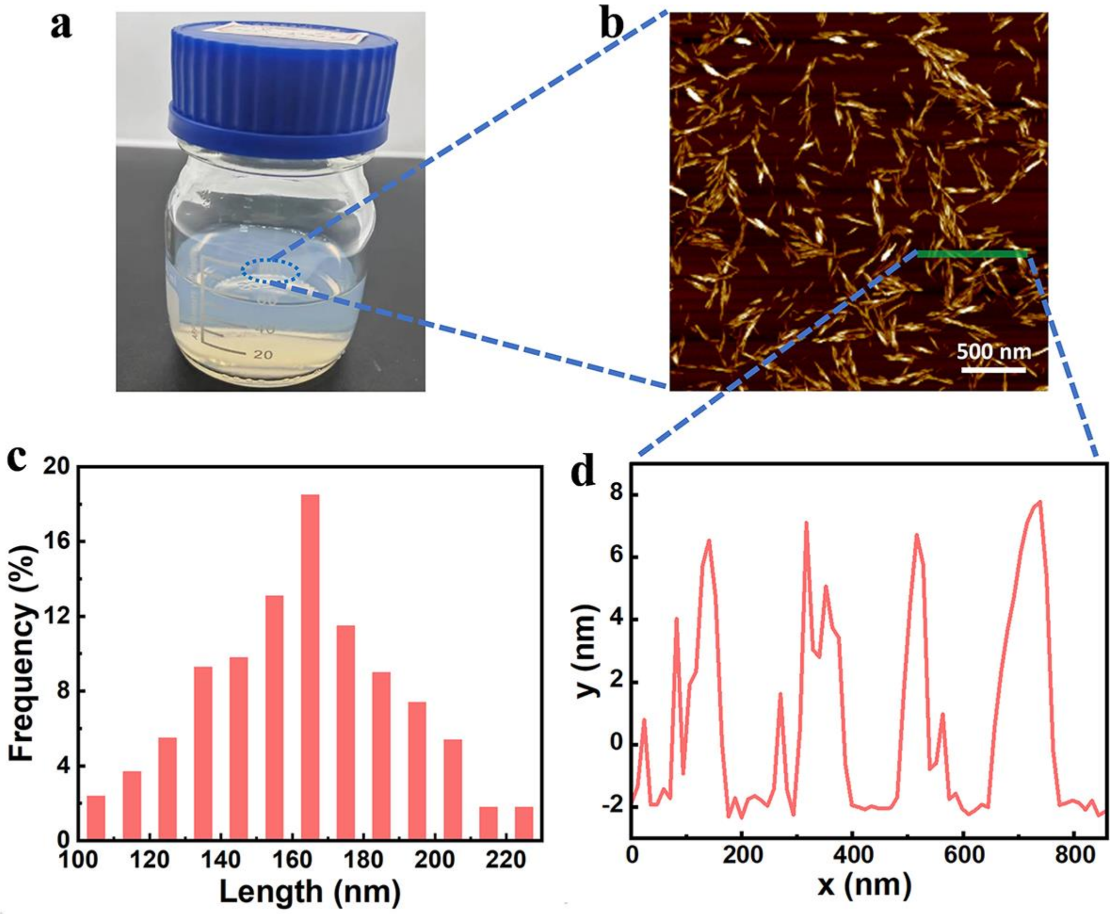

Figure S1. (a) Image of CNC suspension; (b) AFM image of CNC; (c) statistical distribution of length of CNC, and (d) corresponding CNC section profile.

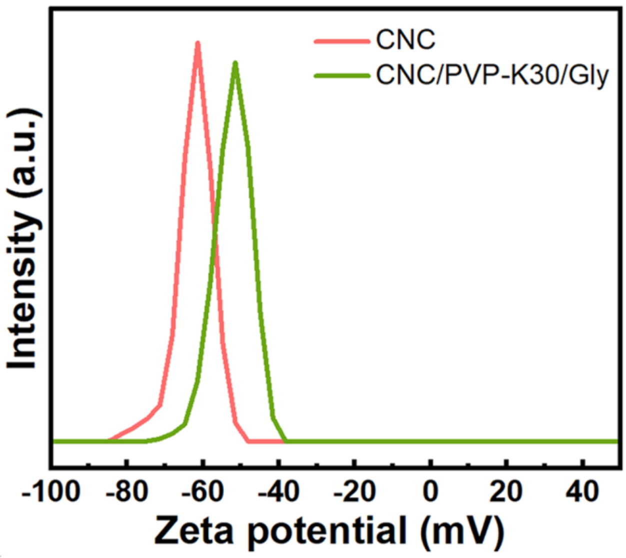

Figure S2. Zeta potential of CNC and CNC/PVP-K30/Gly (CPG) suspension.

The zeta potential of the pure CNC suspension measured approximately -61 mV, while the

CNC/polyvinylpyrrolidone-K30/glycerol (CPG) suspension registered around -51 mV.

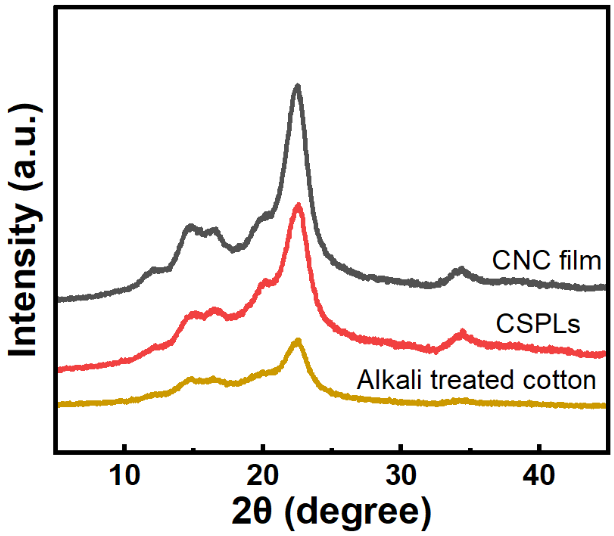

Figure S3. XRD patterns of pure CNC film, CSPLs, and alkali treated cotton.

XRD analysis of CNC film, CSPLs, and alkali-treated cotton paper revealed four identical diffraction peaks. The 2θ positions of these peaks were located at 14.7°, 16.5°, 22.6°, and 34.5° respectively, suggesting that the acid hydrolysis and the addition of PVP-K30 did not alter the crystal structure of CNC.

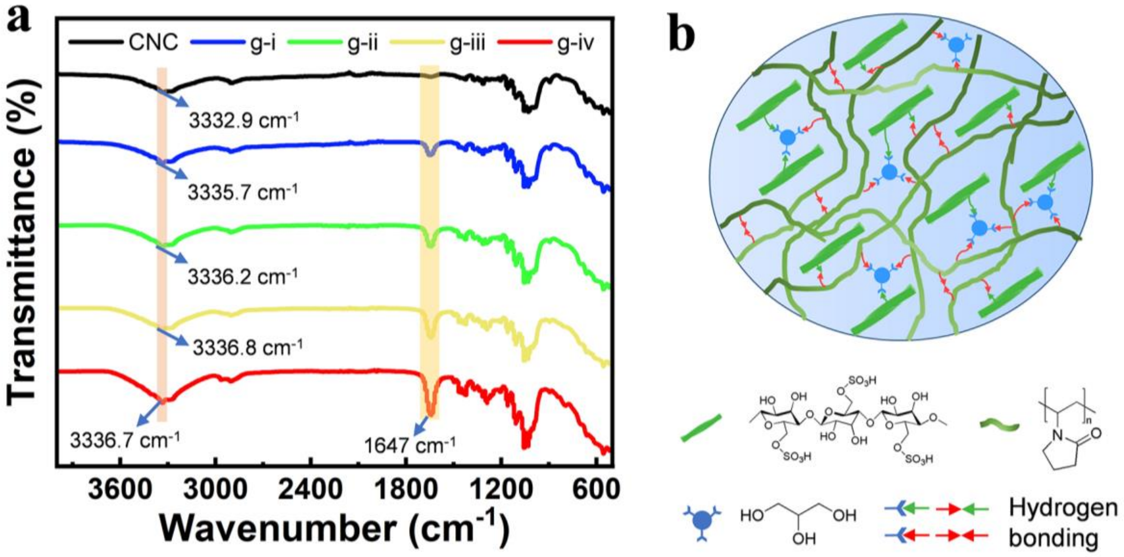

Figure S4. (a) FT-IR spectra of pure CNC film and CSPLs of Figure 2g. (b) Schematic diagram of hydrogen bonds cross-linking network of CSPLs.

The surface of CNC contains numerous hydroxyl groups, which interact with the amide groups in PVP-K30 and the hydroxyl group of glycerol through hydrogen bonding.The pure CNC film

exhibits a band at 3332.9 cm^{-1}, corresponding to the O-H stretching group. In contrast, the CSPLs exhibit a band at around 1647 cm^{-1}, corresponding to the amide bonds. Upon addition of PVP-K30, a slight shift in the band from 3332.9 cm^{-1} to 3335.7 cm^{-1} is observed, indicating a weakening of the hydrogen bond inherent in CNC. This is due to the formation of hydrogen bonds interactions between the amide group and the hydroxyl group of PVP-K30 in the CSPLs. Furthermore, regardless of the amount of PVP-K30 added, the band corresponding to its O-H stretching group remains at approximately 3336 cm^{-1}. This could be attributed to the formation of hydrogen bonds between the added glycerol and CNC and PVP-K30.

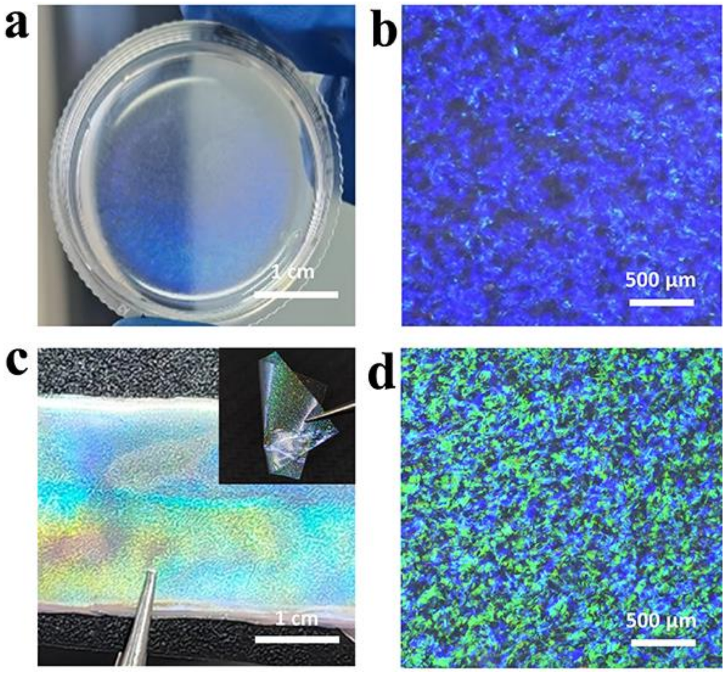

Figure S5. (a) CNC film under naked eye. (b) Optical picture of the CNC film was taken through a stereomicroscope. (c) CSPLs under naked eye. (d) Optical picture of the CSPLs was taken through the stereomicroscope.

When observed under the naked eye, the surface of the pure CNC film reflects a structural color. However, under a stereomicroscope with a black card as a substrate, the surface color distribution appears discontinuous. It primarily consists of blue “islands” ranging from tens to hundreds of microns in size, interspersed with transparent regions. These “islands” are termed SCDs. To enable the coexistence of different colors SCDs on the film surface, a common approach involves introducing a filler medium into the CNC. In this study, PVP-K30 as the principal filler, and Gly were introduced to form extra hydrogen bond to enhance the film's flexibility. Similarly, when the surface of the CSPLs was observed under a stereomicroscope, the SCDs predominantly showed blue and green colors, with transparent and some cyan regions also distributed across on the film surface.

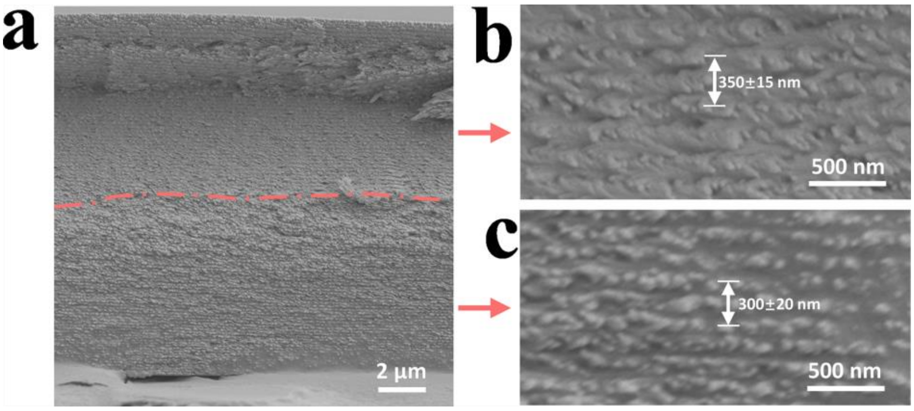

Figure S6. (a) Twist defect in the cross-section of CSPLs film. (b) Localized magnification of the upper layer in (a) with a pitch of 350 ± 15 nm. (c) Localized magnification of the lower layer in (a) with a pitch of 300 ± 20 nm.

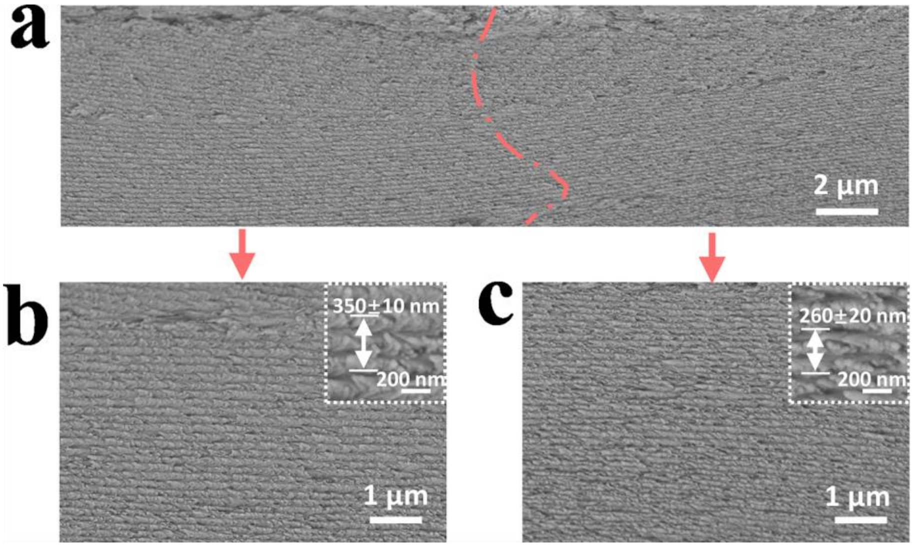

Figure S7. (a) Line defect in the cross-section of CSPLs film. (b) Localized magnification of the left in (a) with a pitch of 350 ± 10 nm. (c) Localized magnification of the right in (a) with a pitch of 260 ± 20 nm.

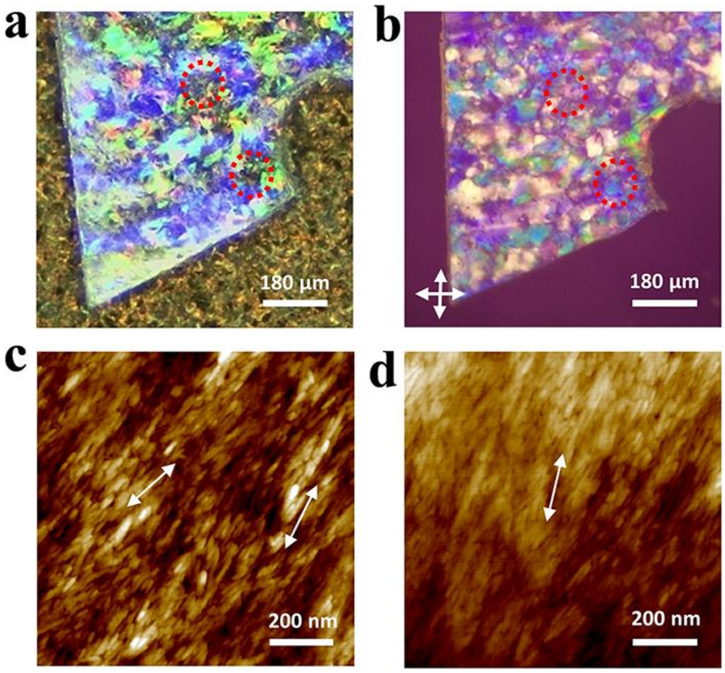

Figure S8. (a) Optical picture of the CSPLs with the transparent region of the film surface labeled by the red circle. (b) POM image of the CSPLs response in (a). (c) AFM image of the color region of the CSPLs. (d) AFM image of the transparent region of the CSPLs.

POM and AFM were employed for comparative characterization of the transparent and colored regions in the CSPLs. As shown by the POM images, the transparent regions of the CSPLs (indicated by the red circle in (b)) exhibit significant birefringence, similar to that in the colored region. In addition, AFM analysis (see (c) and (d)) reveals that both regions exhibit a remarkably similar aligned and ordered morphology. These results indicate the presence of chiral nematic phases on the surface and interior of the transparent region.

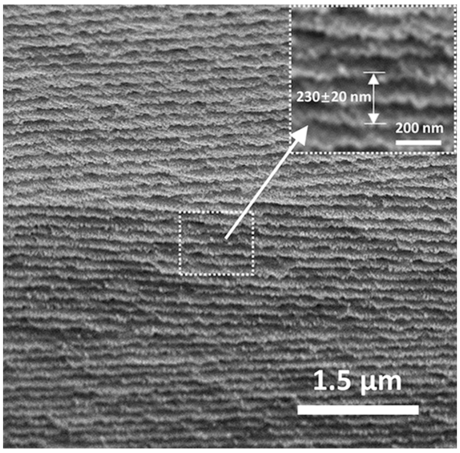

Figure S9. SEM image of the corresponding cross-section of the transparent region.

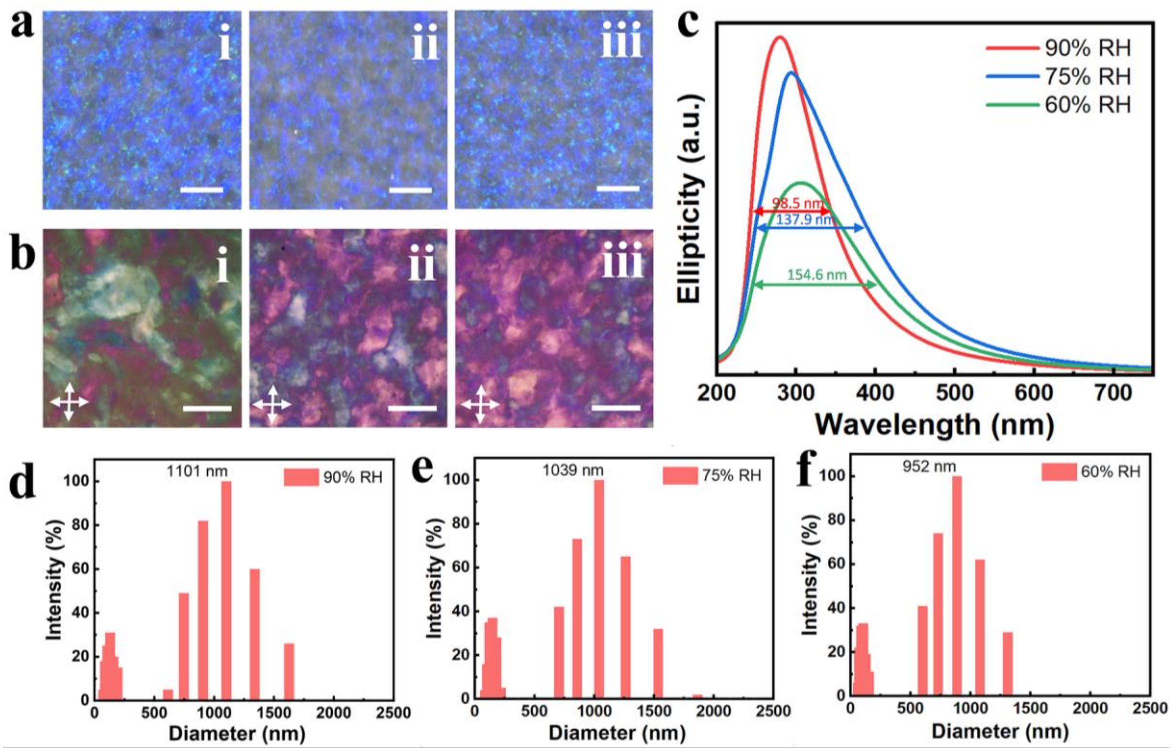

Figure S10. (a) Images of CNC films (scale bars, 500 μm) obtained in an evaporative environment at 20 °C and relative humidities of i) 90% RH, ii) 75% RH, and iii) 60% RH, respectively, and (b) POM images (scale bars, 100 μm) of the corresponding CNC films in (a) taken with a cross-polarizer. (c) Circular dichroic spectral of the corresponding CNC films in (a). (d-f) Statistical plots of particle size distribution after evaporation of the suspension mass fraction from 3.2 wt.% to 4.2 wt.% at 20 °C and relative humidity of 90%, 75% and 60%, respectively.

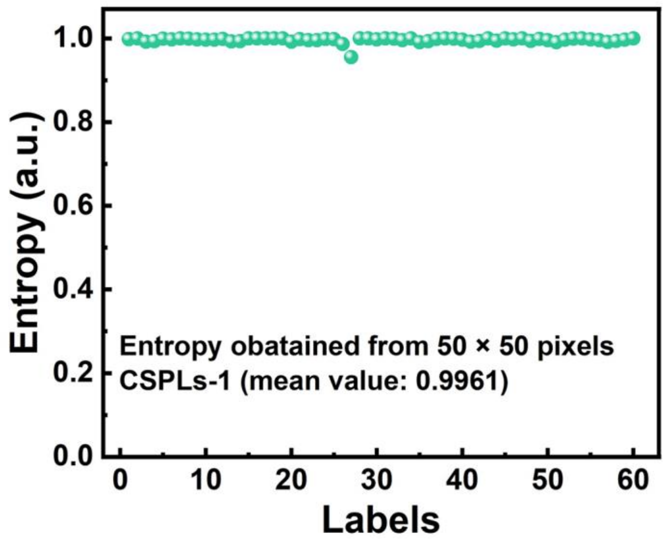

Figure S11. The entropy of 60 50 × 50 pixels CSPLs-1. Entropy calculations for the 60 50 × 50 pixels CSPLs-1 show values close to the ideal 1, with an average entropy of 0.9961 and a minimum entropy of 0.9905.

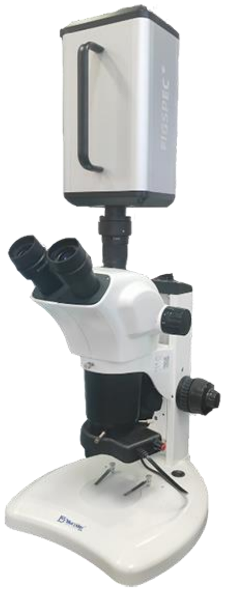

Figure S12. Hyperspectral imaging system (HIS), consisting of a stereomicroscope and a hyperspectral camera.

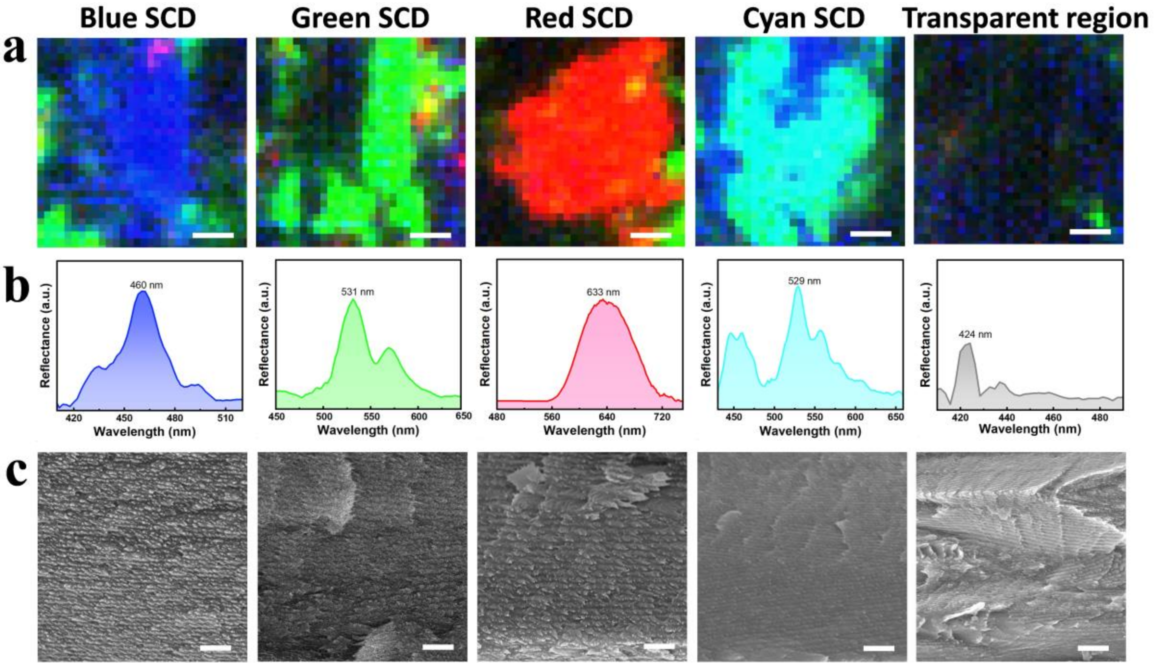

Figure S13. (a) Images of SCDs in different colors (scale bars 25 μm), corresponding reflected light spectra extracted using the HIS shown in (b). SEM images of cross-sections in the corresponding areas of (a) are shown in (c), the scales from left to right are 1μm, 1μm, 1μm, 1.5μm and 4 μm, respectively.

The spectral positions corresponding to the highest peaks of the reflectance spectra for the blue SCD, green SCD, and red SCD are 460 nm, 531 nm, and 643 nm, respectively. The corresponding pitches for these SCDs are approximately 301 nm, 350 nm, and 420 nm, as evidenced by the cross-section SEM images in (c). Cyan SCDs exhibit dual reflection peaks at

460 nm and 529 nm, reflecting both blue and green light spectral peaks, a characteristic more pronounced in cyan SCDs compared to other SCDs (see (a)). SEM analysis reveals that the cross section of cyan SCDs comprises domains reflecting blue light (with a pitch of approximately 230 nm) and green light (with a pitch of about 349 nm, shown in (c)). For the transparent region, its reflectance spectra show a weak peak at 424 nm.

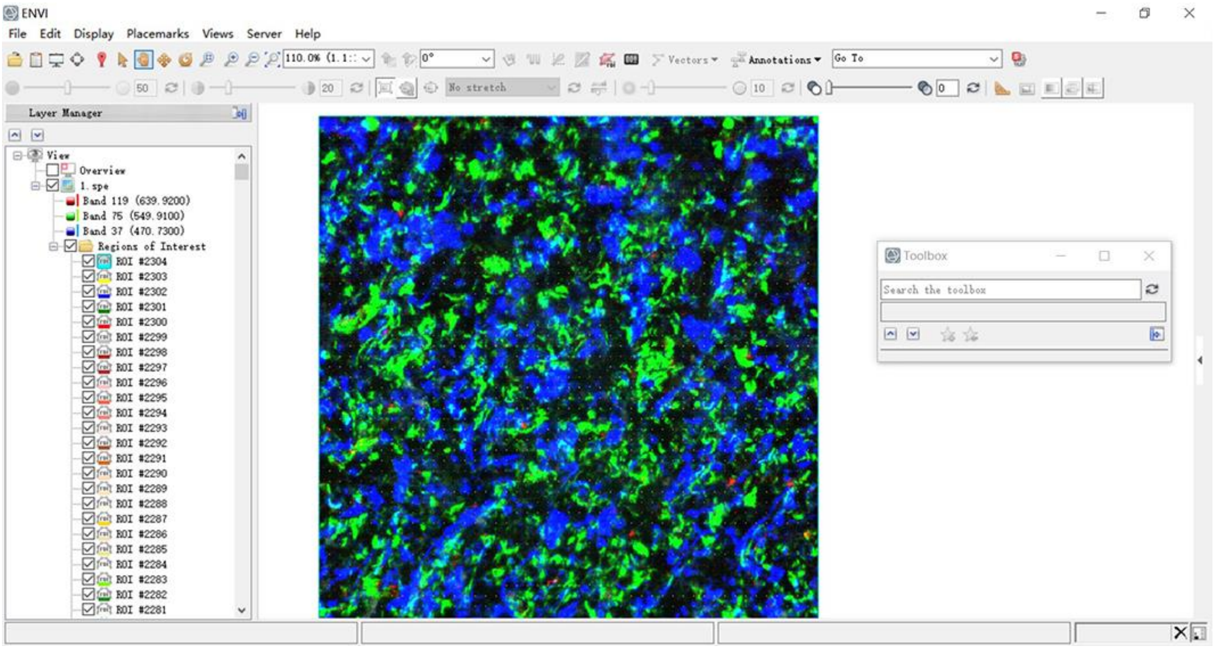

Figure S14. The ENVI software establishes 48 × 48 bit regions of interests (ROIs) to gather the reflectance spectral data from the labels.

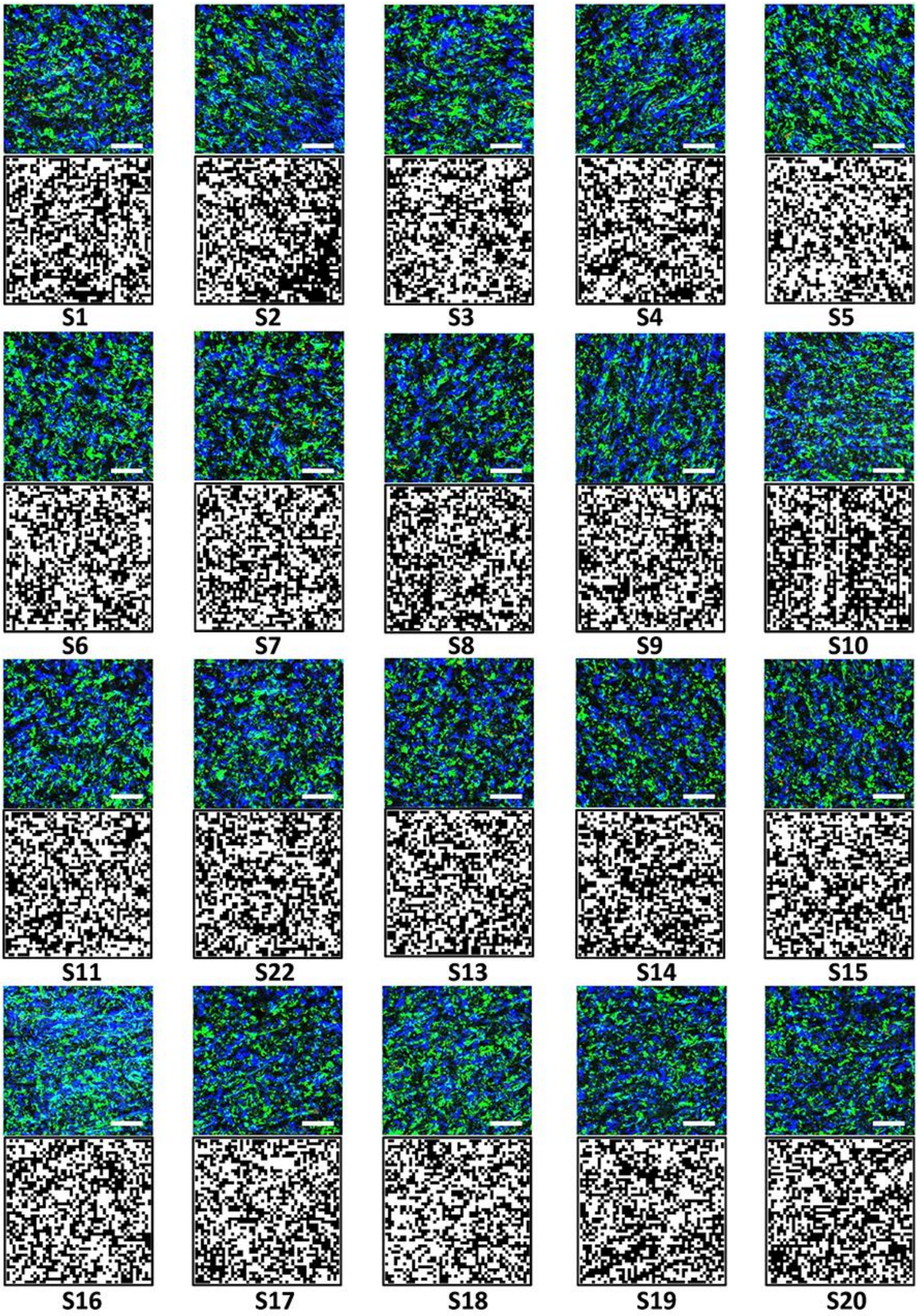
S1
S2
S3
S4
S5
S6
S7
S8
S9
S10
S11
S22
S13
S14
S15
S16
S17
S18
S19
S20

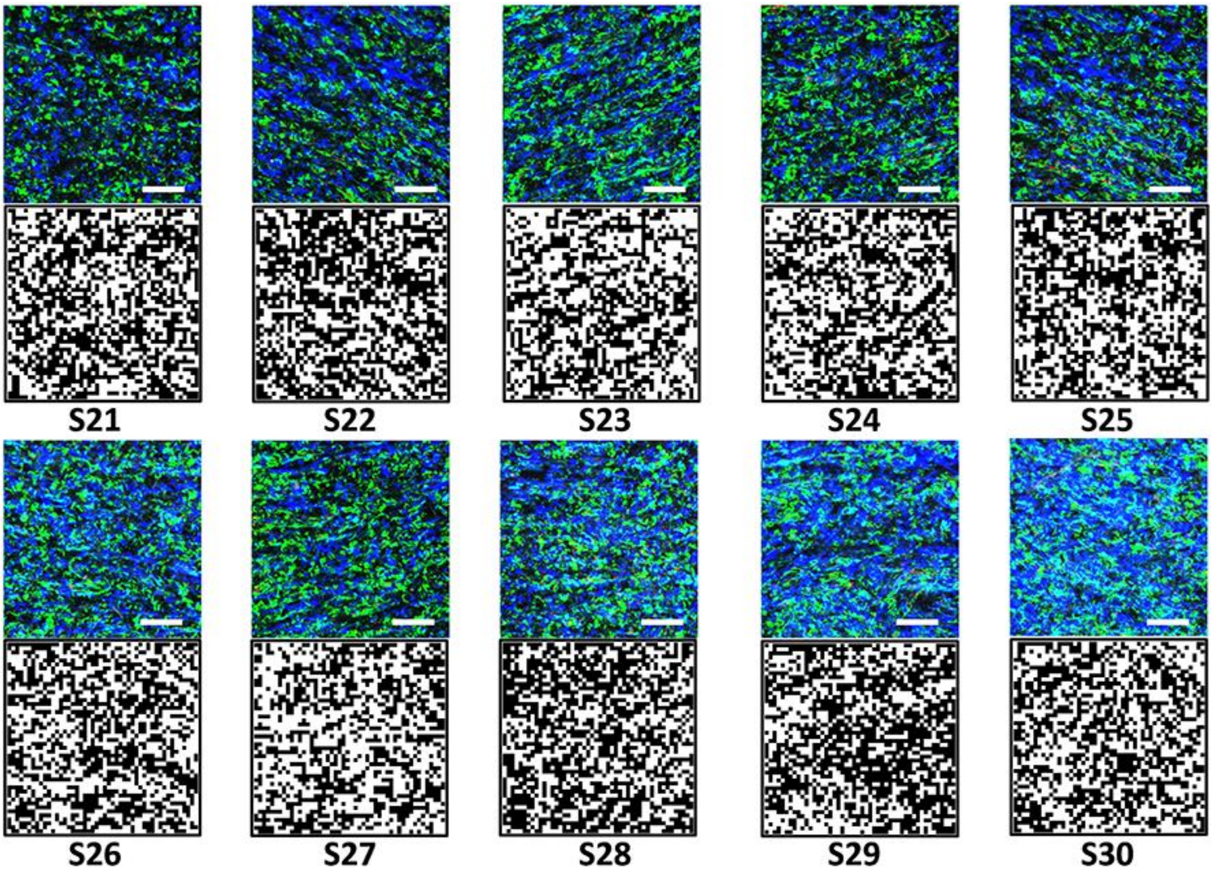

Figure S15. 30 images of CSPLs-2 keys (48 × 48 pixels, scale bars, 500 μm).

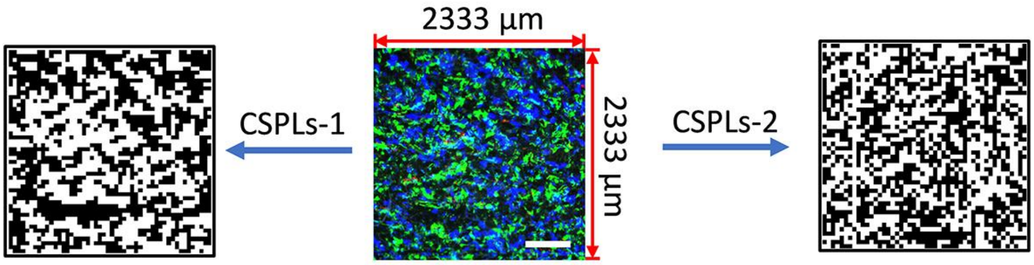

Figure S16. 50 × 50 pixels CSPLs-1 key and 48 × 48 pixels CSPLs-2 key for the same label (scale bars, 500 μm).

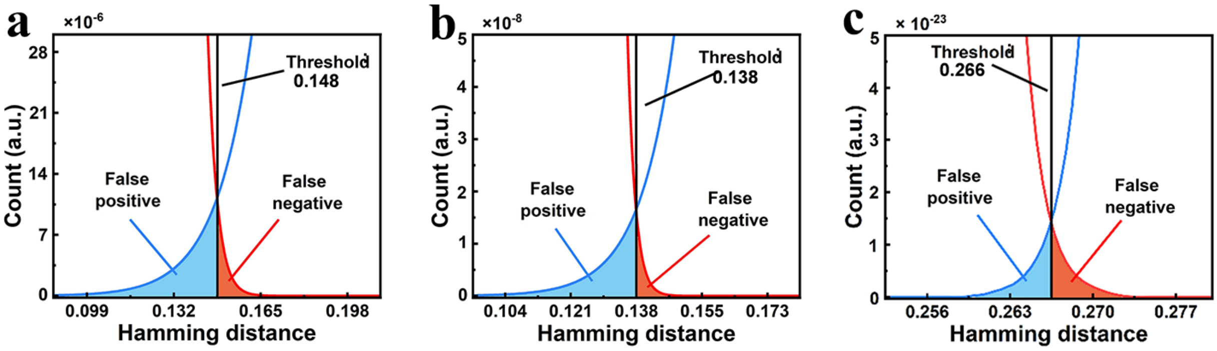

Figure S17. Intra HD and inter HD for authentication thresholds of (a) 20 × 20, (b) 30 × 30,

and (c) 48 × 48 pixels CSPLs-2 keys.

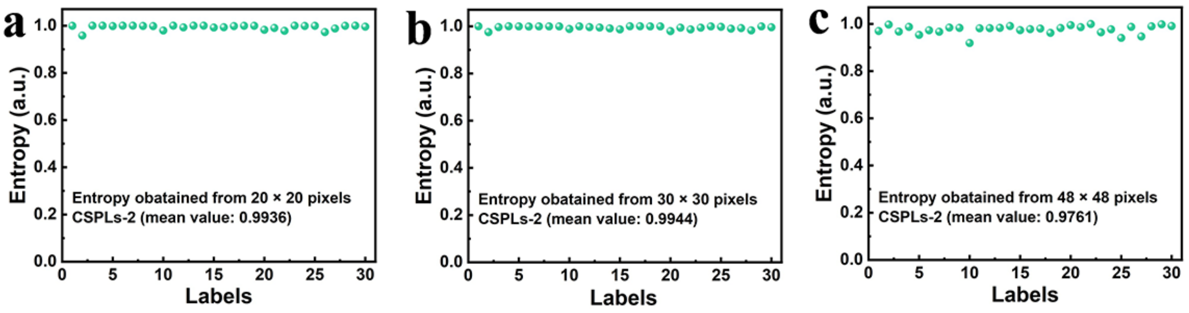

Figure S18. The entropies of 20 × 20, 30 × 30 and 48 × 48 pixels CSPLs-2 keys.

Table S1. NIST randomness test of binary sequences (12000-bit) generated from 30 different 20 × 20 pixels CSPLs-2

NIST statistical test	*p*-value	Result
Frequency	0.042706	Success
Block frequency	0.063118	Success
Rank	0.385467	Success
Runs	0.120798	Success
Cumulative sums	0.061005	Success
Longest run of ones	0.773411	Success
Non-overlapping template matching	0.023627	Success
Linear complexity	0.121129	Success
Serial	0.695002	Success

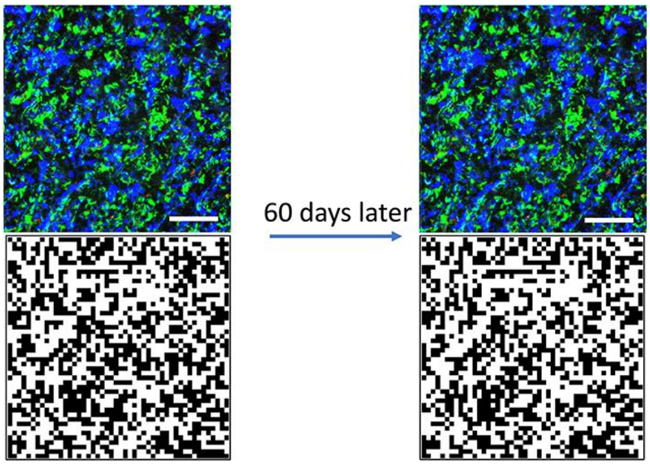

Figure S19. A label and its corresponding 48 × 48 pixels CSPLs-2 key were examined after 60 days in a regular environment in comparison to their initial state (Scale bars, 500 μm).

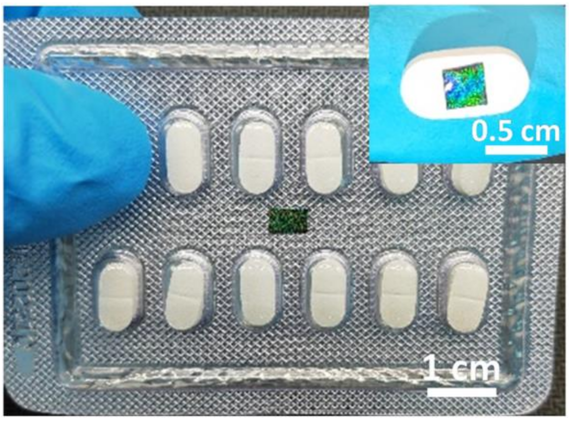

Figure S20. PUF label attached to pharmaceutical product.

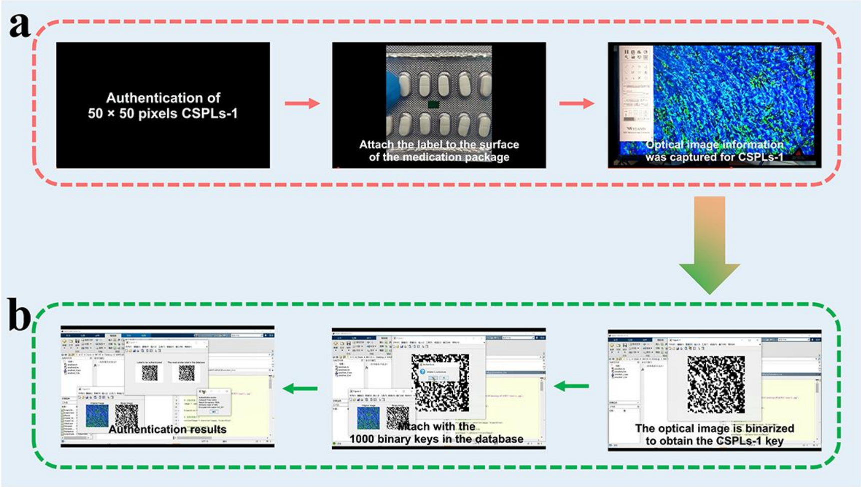

Figure S21. CSPLs-1 key authentication process. (a) An optical picture of the target area in the label was taken using a stereomicroscope. (b) The key was obtained by binarizing the optical image through MATLAB and uploaded to the database for matching.

Table S2. Comparison of this work with the reported main parameters of optical mapping

Optical mapping	Processing pixel size	processing time	Refs
Raman mapping	50 × 50 pixels	20 min	1
Raman mapping (Confocal Raman microscopy)	480 × 480 pixels	≈ 230 seconds	2
Structural color mapping	50 × 50 pixels	≈ 127 min	3
Fluorescence mapping	480 × 480 pixels	≈ 460 seconds	4
Fluorescence mapping	480 ×480 pixels	≈ 34 - 69 seconds	5
This work	480 × 480 pixels	≈ 5 seconds	—

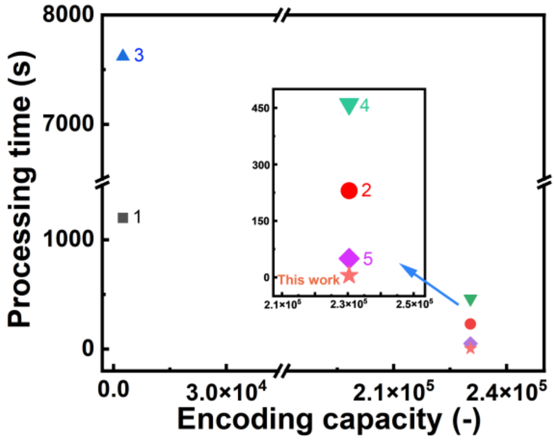

Figure S22. Comparison of the main parameters based on Table S2.

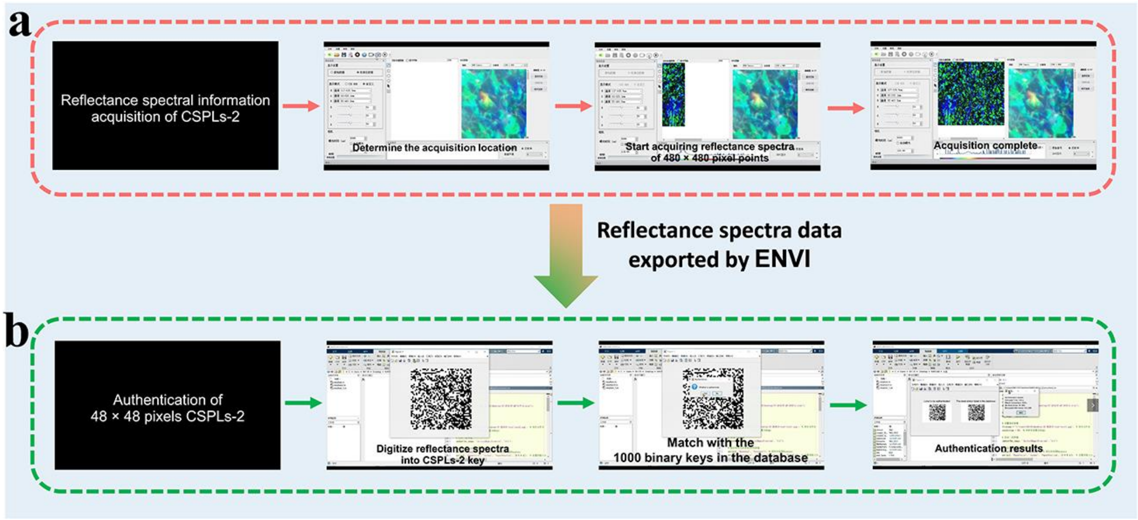

Figure S23. CSPLs-2 key authentication process. (a) The optical picture of the label was taken using the HIS, and the reflectance spectral information of all pixels was obtained at the same time. (b) After the reflectance spectral information was exported by ENVI, the spectral information was digitized by MATLAB to get the key and uploaded to the database for matching.

Table S3. The time required for the database construction and authentication process

Size of CSPLs-2 key	Mapping of HIS	Reading of spectral information in ENVI	Matching in MATLAB
20 × 20 pixels	5 seconds	38 seconds	0.5 ~ 0.7 second
30 × 30 pixels	5 seconds	89 seconds	0.5 ~ 0.7 second
48 × 48 pixels	5 seconds	5 minutes	0.5 ~ 0.7 second

Table S4. Comparison of this work with already reported authentication matching times.

PUF type	Coding capacity	processing time (Database size)	Refs
Structural color	10^{100}	2 seconds (1000 labels)	3
Raman	$2^{4\times10\,000}/(100\ \text{pixels})^{2}$	28 seconds (Not available)	6
Localized surface plasmon resonance	10^{243}	120 seconds (Not available)	7
Polymer wrinkle	1×10^{135}	≈ 40 min (1300 labels)	8,9
This Work	2^{2500} (CSPLs-1) & 2^{2304} (CSPLs-2)	0.5-0.7 second (1000 labels)	—

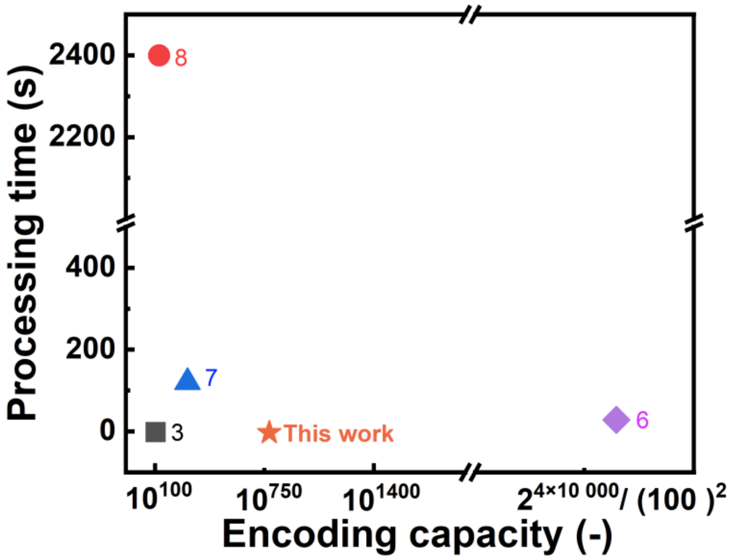

Figure S24. Comparison of the main parameters based on Table S4.

References

[1] Y. Gu, C. He, Y. Zhang, L. Lin, B. D. Thackray, J. Ye, *Nat. Commun*. **2020**, *11*, 516.
[2] S. Nair, J. Gao, Q. Yao, M. H G Duits, C. Otto, F. Mugele, *Natl. Sci. Rev.* **2020**, *7*, 620-628.
[3] J. Wu, X. Liu, X. Liu, Z. Tang, Z. Huang, W. Lin, X. Lin, G. Yi, *Chem. Eng. J.* **2022**, *439*, 135601.
[4] C. Kraft, H. Hempel, V. Buschmann, T. Siebert, C. Heisler, W. Wesch, C. Ronning, *J. Appl. Phys*. **2013**, *113*, 124510-124518.
[5] C. G. Ryan, D. P. Siddons, R. Kirkham, Z. Y. Li, M. D. de Jonge, D. Paterson, J. S. Cleverley, A. Kuczewski, P. A. Dunn, M. Jensen, G. De Geronimo, D. L. Howard, B. Godel, K. A. Dyl, L. A. Fisher, R. H. Hough, Stephen J. Barnes, P. A. Bland, G. Moorhead, S. A. James, K. M. Spiers, G. Falkenberg, U. Boesenberg, G. Wellenreuther, *The Maia detector array and X-ray fluorescence imaging system: locating rare precious metal phases in complex samples.* SPIE, Bellingham, **2013**, pp. 88510Q–88510Q.
[6] H. Guo, Y. Oin, Z. Wang, Y. Ma, H. Wen, Z. Li, Z. Ma, X. Li, J. Tang, J. Liu, *Adv. Funct. Mater.* **2023**, 2304648.
[7] J. D. Smith, M. A. Reza, N. L. Smith, J. Gu, M. Ibrar, D. J. Crandall, S. E. Skrabalak, *ACS Nano* **2021**, *15*, 2901-2910.
[8] H. J. Bae, S. Bae, C. Park, S. Han, J. Kim, L. N. Kim, K. Kim, S. H. Song, W. Park, S. Kwon, *Adv. Mater.* **2015**, *27*, 2083.
[9] L. Jing, Q. Xie, H. Li, K. Li, H. Yang, P. L. P. Ng, S. Li, Y. Li, E. H. T. Teo, X. Wang, P. -Y. Chen, *Matter* **2020**, *3*, 2160.
